# Influences on Participation in Life After Spinal Cord Injury: Qualitative Inquiry Reveals Interaction of Context and Moderators

**DOI:** 10.3389/fresc.2022.898143

**Published:** 2022-05-31

**Authors:** Delena Amsters, Melissa Kendall, Sarita Schuurs, Pim Kuipers

**Affiliations:** ^1^Spinal Outreach Team, Queensland Spinal Cord Injuries Service, Metro South Health, Brisbane, QLD, Australia; ^2^The Hopkins Centre, Menzies Health Institute, Griffith University, Brisbane, QLD, Australia; ^3^Transitional Rehabilitation Program, Queensland Spinal Cord Injuries Service, Metro South Health, Brisbane, QLD, Australia; ^4^Central Queensland Centre for Rural and Remote Health, James Cook University, Emerald, QLD, Australia

**Keywords:** spinal cord injury, participation, community integration, qualitative inquiry, literature review, thematic synthesis, rehabilitation theory

## Abstract

**Background:**

Greater understanding of the influences on participation in life after spinal cord injury (SCI) can inform rehabilitation theory and practice. Careful qualitative inquiry can reveal subjective meanings associated with the relevant experiences, strategies, and perceptions of those with lived experience of SCI. A search of literature, followed by a thematic synthesis of qualitative studies, was undertaken to bring together these insights in a meaningful way.

**Methods:**

The research question guiding the literature review and synthesis was, *What do people with SCI perceive to be the influences on their participation in life*? Three critical databases were searched for qualitative studies examining influences on participation in life after SCI. Peer-reviewed studies published after 2006, involving adults with SCI living in countries with advanced economies, were included. Data were extracted from 24 articles and subjected to three-level thematic synthesis—the coding of primary data from the studies, the development of descriptive themes based on an organization of those codes, and the generation of analytical themes.

**Results:**

The synthesis yielded five analytical themes, supported by 17 descriptive themes. The analytical themes were (1) external contextual influences, (2) personal physical context, (3) personal psychological context, (4) potential moderators of participation outcomes, and (5) temporal dimensions of participating in life after SCI.

**Interpretation:**

These themes highlight the complex interactions that shape participation from the perspective of people with SCI. Closer examination of the potential moderators may provide insights into effective rehabilitation interventions.

**Conclusions:**

Synthesis of qualitative inquiry provides valuable insights into the perceptions of influences on participation in life from the point of view of people with SCI. The findings of this synthesis are instructive for rehabilitation theory and practice. It can complement what we learn from using the ICF to understand participation.

## Background

Participation is a dimension of human functioning, described in the World Health Organization's International Classification of Functioning, Disability and Health (ICF) as involvement in a life situation (1). Despite the simplicity of this definition, participation is recognized as a highly complex construct (2). The term *participation* is often used holistically to capture those aspects of human functioning that relate to socially defined tasks and roles (3, 4). This encompasses, but is not limited to, creating and maintaining interpersonal relationships, assisting or caring for others, undertaking education, working for remuneration or volunteering, partaking in recreation or leisure pursuits, and engaging in civic activities (1).

Participation is depicted in the ICF as one of the interactive components of functioning—along with body functions and structures, and activities. All components are subject to the impacts of heath conditions, environmental factors and personal factors (5). As such, the ICF reflects a biopsychosocial approach to understanding the multiple, complex, and dynamic influences on human functioning, in the presence of health disorders or disease (6).

When examining participation from the perspective of people with spinal cord injury (SCI), each individual's understanding of what is meant by the term is likely to differ based on personal experiences and context (7, 8). As well as being seen as a term for describing the regular roles and tasks of life, some people with SCI also equate participation with such things as sense of autonomy, having choices in life and experiencing societal inclusion (9). Conceptualizing and measuring participation are important steps toward defining clinical outcomes for people with spinal cord injury (SCI), however developing an understanding of what influences participation is likely to contribute directly to enabling them to attain these outcomes. Recognizing the influences on participation in life after SCI can also inform rehabilitation theory and practice (10).

The International Classification of Functioning, Disability and Health (ICF) is the pre-eminent taxonomy for describing human functioning (including participation in life situations) in the presence of health conditions (11). The potential influences on participation recognized by the ICF are extremely broad in scope—covering personal characteristics, environmental variables, and impairments of body structure and function. The ICF provides rehabilitation practitioners with a structure within which to assess the function of a person with SCI (5). However, while the ICF structure classifies key dimensions, it does not reveal the full breadth and complexity of influences on participation in life after SCI.

Qualitative inquiry provides a means of exploring influences on participation in life after SCI. While the ICF codifies barriers to and facilitators of participation, qualitative approaches can add additional nuance by revealing subjective meanings underlying the experiences, strategies, or perceptions of those with lived experience of SCI.

In 2018 we published a study about determinants of participation in life after SCI. These determinants were proposed after examination of the narratives of allies with SCI (12). That work was preceded by a comprehensive mapping exercise of both qualitative and quantitative literature about participation in life after SCI (13). The current article presents an up-to-date literature search (December, 2021) and thematic synthesis of one component of literature identified in that map, namely peer reviewed qualitative studies relating to, or describing, influences on participation in life after SCI. The purpose of this targeted search and thematic synthesis is to continue to build our understanding of the lived experience of participation in life after SCI, to further inform rehabilitation theory and practice.

## Methods

### Review Question

Our specific review question *was (using the lens of published research): What do people with SCI perceive to be the influences on their participation in life?* The spider approach (sample, phenomenon of interest, design, evaluation, and research type) was used to develop a search strategy to inform this question (14). The sample was *people with SCI*, the phenomenon of interest was *participation in life*, the design of studies was *interviews or other methods which elicit personal perceptions*, the construct under evaluation was *influences on participation*, and research type was *qualitative*.

### Literature Search

A mapping approach to literature review facilitates a comprehensive overview of a body of literature, which can form the basis for targeted explorations to answer specific questions (15). The previous literature mapping exercise (13) informed the search strategy for the current review, with the addition of terms to narrow the search to articles about qualitative studies. CINAHL, Medline, and PsycInfo databases were searched using the search strings shown in Supplementary Material 1.

Articles considered within scope were those which described studies examining one or more influences on participation for people with adult-onset SCI, published between 2006 and 2021 (inclusive). Only peer reviewed journal articles that presented primary qualitative research were included. Articles presenting mixed methods work were included if the qualitative component reported direct quotations and clear paraphrasing of participant responses. Opinion pieces, editorials, and reviews were considered outside scope.

A risk in secondary analysis of qualitative data is de-contextualizing findings (16). Therefore, to optimize transferability across health and social care systems, our search was limited to studies conducted in countries with highly developed economies (17) with a predominant “Western” culture.

The searches yielded 522 unique articles. Article screening was managed using COVIDENCE^TM^ software. Two reviewers independently screened each article for relevance, by reviewing abstract and title. Contentious articles were discussed, until consensus was reached. The full texts of the remaining 110 articles were independently examined by two reviewers. Where reviewers were not in agreeance on inclusion, it was sent to a third reviewer for adjudication. Articles were excluded at this stage of screening if the studies they described either *failed to examine influences on participation* or made minimal mention of influences on participation and would thus have *limited capacity to inform the synthesis*.

Based on the overview provided by the mapping exercise, articles which primarily examined employment, recreation and leisure, or interpersonal relationships, rather than participation or community integration, were excluded. As the search strings did not explicitly include *employment, recreation, and leisure* or *interpersonal relationships*, this could not be considered a comprehensive literature search of these domains, so it was agreed to exclude them from the final list of articles for this current synthesis.

**Figure 1:**
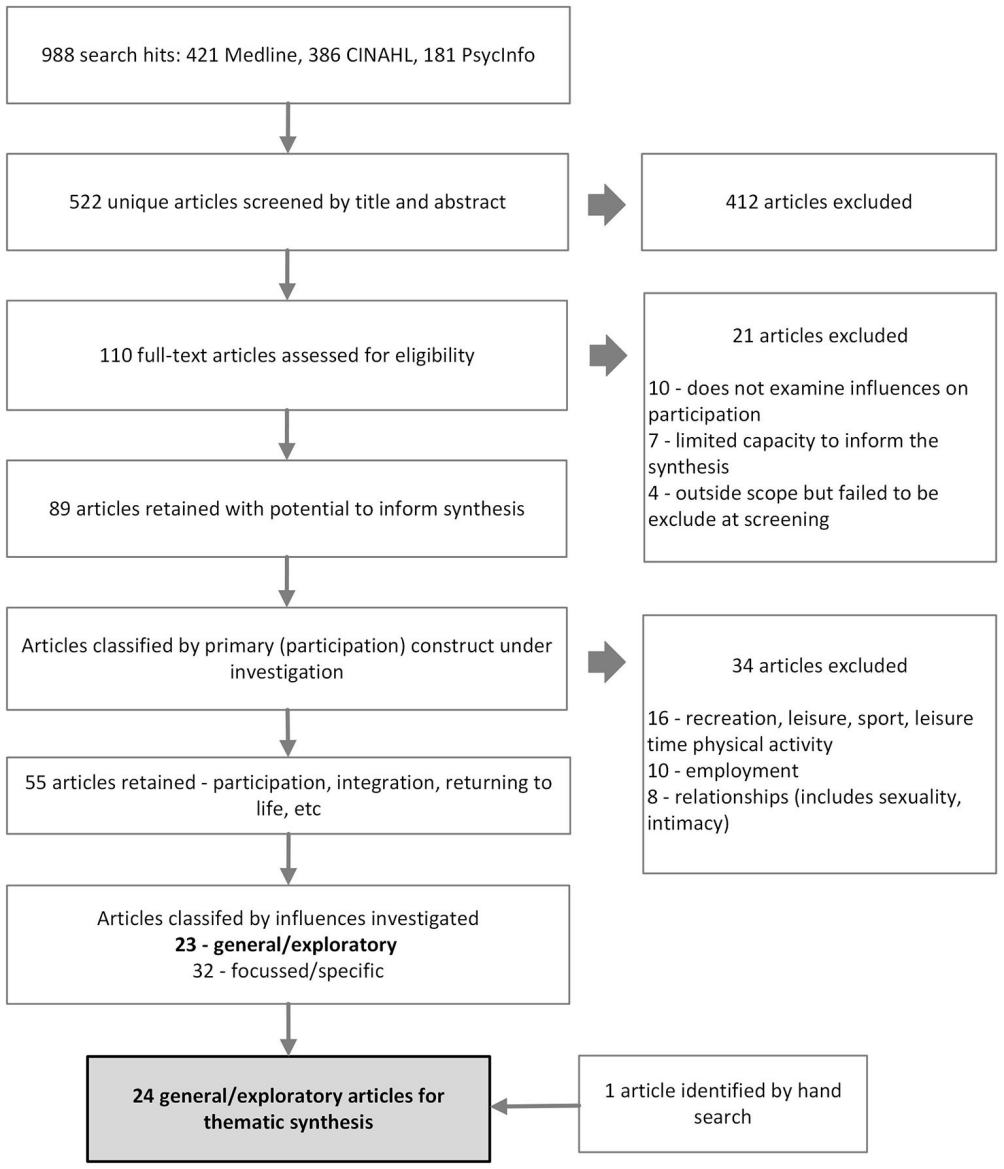
Process of literature search and screening.

The tally of articles within the scope of the topic and relevant to the research question was 55. This comprised 23 primary articles with a general or exploratory approach to understanding participation and 32 secondary articles which had a particular focus or addressed a specific issue associated with participation (Figure 1). The decision was made to exclude these 32 secondary articles, since their focus was on specific topics such as the influence of aging, or physical activity or pain relief, which might confound the synthesis. However, a list of these articles and their attributes can be found in Supplementary Material 3.

Finally, hand searching of reference lists of the included articles was also undertaken. This resulted in one further article, taking the final tally to 24. The search, selection and screening process is summarized by Figure 1.

### Study Attributes

Basic attributes were recorded for each of the 24 studies which met the inclusion criteria. Attributes included:

- Country in which the study was conducted
- Purpose of the study
- Data collection methods
- Method of data analysis
- Target demography (e.g., people with non-traumatic etiology)
- Number of participants
- Inclusion of participants other than people with SCI

Recognizing that formal quality appraisal of such studies is of limited value (16), we noted that restricting our synthesis to peer reviewed studies cataloged in the three principal databases for disability and rehabilitation literature was a suitable proxy for baseline quality.

### Thematic Synthesis of Reported Data

The results sections of the primary studies (24 articles) categorized as general/exploratory, were distilled via the thematic synthesis approach of Thomas and Harden (16). To ensure precision and rigor of our synthesis, we considered only the *Results* section of studies as containing data for coding. Further, to remain as close as possible to the “voice” of people with SCI, we limited our analysis to direct quotes and instances of explicit paraphrasing from primary data. We avoided including interpretive content. Coding of units of meaning (most frequently sentences or paragraphs) was based on manifest content. Codes were often labeled using words or phrases from the studies themselves. All data were initially coded by one reviewer.

This approach had three stages: coding of all relevant direct quotes from the results sections of each of the studies; the development of descriptive themes based on organization of those codes; and the generation of analytical themes that propose new interpretations of the data. Descriptive themes emerged during the coding process and were refined at the conclusion of coding. Eighteen themes emerged after condensing similar codes and clustering related codes. Trustworthiness of coding and theme generation was determined by performing a backwards audit. This was conducted by two reviewers. They were tasked with checking themes against the original data set. They also assessed each theme for internal coherence, consistency, and distinctiveness (18). This backwards audit process resulted in collapsing two descriptive themes into one, renaming of one theme, a secondary search for data to support the continued inclusion of one theme, and the moving of some exemplar quotes between themes.

Finally, the remaining 17 descriptive themes were considered from an analytical or interpretive perspective with reference to the original research question. All reviewers participated in shaping the analytical themes.

## Results

The studies described in 24 qualitative research articles were examined to inform the answer to the research question *What do people with SCI perceive to be the influences on their participation in life*? Articles were from Australia (*n* = 5), United States of America (*n* = 4), Belgium (*n* = 3), United Kingdom (*n* = 2), Canada (*n* = 2), Sweden (*n* = 2), as well as single articles from a variety of European countries. Study sample sizes ranged from one to 54 participants. The total number of participants across articles was 352. However, studies may be reported across multiple articles therefore each article may not represent a unique set of participants.

Semi-structured interviews with thematic analysis was the most frequent method of data collection and analysis. Table 1 provides a summary of the attributes of the studies described in the 24 articles. Details of the attributes of each study can be found in Supplementary Material 2.

**Table 1:** Summary of attributes of articles in synthesis (*n* = 24).

**Attribute**		**No**.
Country in which study was set	Australia	5
	Belgium	3
	Canada	2
	Croatia	1
	Denmark	1
	Mixed European	1
	Netherlands	1
	New Zealand	1
	Sweden	2
	Switzerland	1
	UK	2
	USA	4
		
SCI participants only	Yes	22
	No	2
Target demography	Yes (e.g., non-traumatic etiology)	10
	No	14
Data collection methods	Semi structured interviews	17
	Unstructured interviews	1
	Open survey questions	1
	Multiple methods (e.g., interviews and focus groups)	5
Principal approach to analysis	Thematic analysis	6
	Constant comparison	5
	Grounded theory	3
	Content analysis	3
	Phenomenological analysis	3
	Narrative analysis	2
	Other	2

**Figure 2:**
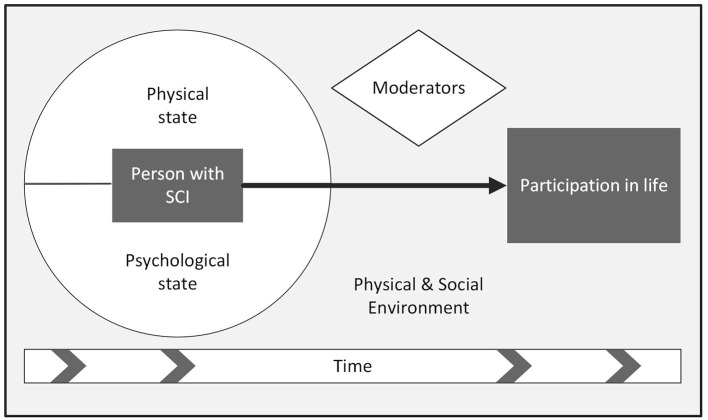
Interconnected elements that influence participation after SCI.

The analysis resulted in the identification of 17 descriptive themes. Consideration of these themes with respect to the research question guided the creation of five overarching analytical themes. These analytical themes were (A) *the external contextual influences*, (B) *the personal physical state*, (C) *the personal psychological state*, (D) *the potential moderators of participation outcomes*, and (E) *the temporal dimensions of participating in life after SCI*. We labeled these analytical themes collectively as the *interconnected elements that influence participation after SCI* (see Figure 2).

### The External Contextual Influences

The first analytical theme, (A) *external contextual influences*, encompassed two descriptive themes—(A1) *the physical environment will always pose challenges* and (A2) *society (people) can help or hinder*. Representative quotes for each theme are presented in Table 2A.

**Table 2:** Analytical themes and descriptive themes with representative quotes.

**Representative quote**	**References**
**(A) The external contextual influences**	
**A1. The physical environment will always pose challenges**	
“Here's a new estate that's just been built. If I go into that estate, I have to stay on the bitumen road, because the gutters are too steep, I can't get up the gutters, and there is no footpath, it's just grass”	(33)
“In my life, there will never be everything as it should be… so that when you leave the house you do not have to think about anything [barriers], but you can really do the things you want to do”	(24)
“So [unnamed city] is wheelchair accessible. It is so much easier to get around in a wheelchair in [unnamed city] than it is here, in [unnamed city]. So, that was one of my biggest challenges, learning to get around”	(29)
One participant in a manual chair reported that by the time he transferred and dismantled his chair, he was “soaked” or “overheated”	(23)
**A2. Society (people) can help or hinder**	
“Yeah, everybody wants to help somebody in a wheelchair. At least that's what it seems to me”	(31)
“When they see people in a wheelchair it's a stigma (. . .). When they look at me, I just want them to see me. Forget the wheelchair, just see me”	(30)
“It's the moment when people don't notice that you sit in the wheelchair anymore, but they take you as a colleague or somebody who takes part in a discussion”	(30)
“There are things you have a right to and I think it's wrong to have to fight for them, it's hard enough as it is anyway. It breaks you down”	(28)
**(B) The personal physical state**	
**B1. The functional capabilities of an impaired body**	
“… for example, I need to do bowel care every two days and it's difficult to find a hotel room or something like that or being with somebody that doesn't really know how that process works”	(36)
“If I want to go out to a night club or whatever, I have to rely on friends help me into bed which isn't ideal”	(9)
Body functions and structures were rarely mentioned in a positive sense, and if so primarily in terms of still having some function. David, for example, referred to sexual functions: “I realized that I get an erection […] and then it worked.”	(30)
VF made the difficult decision to change career paths away from bench science as she could foresee a situation where she would not be able to perform actions independently due to her impaired hand function.	(12)
**B2. Managing health and secondary conditions**	
“It's pacing yourself. I just can't do day after day. That's why I've decided I just ... haven't got the energy to work anymore, as well, in paid work”	(19)
It's embarrassing for me, but for the people around as well...more so with bowel accidents…but it's the frustration … got your clothes on, you've got into town, your just about to get into the chair and..… you've got to go home”	(23)
…Tom, who described how his daily routine, travel arrangements and selection of AT are highly driven by his desire to prevent pressure ulcer development	(32)
“The care for this wreck here [referring to her body] is just horribly time-consuming. […] I need 3–4 h to get out of the house, depending how it's going with emptying my bowel. […] So I have only very little time left for social relations. Little to no time at all”	(30)
**(C) The personal psychological state**	
**C1. Personal feelings, attitudes, and responses**	
“I always had the confidence that no matter what the circumstances were I had value as a person, regardless of being in a wheelchair. . . . I had enough confidence in myself that I could still accomplish things and do things that would give value to myself, and my life around me, and the people around me. So I could still go and work . . . and build a company . . . and be independent”	(25)
“I don't feel that I have many more problems than a non-injured person; on the other hand, maybe I have somewhat different problems to deal with instead”	(28)
“You can do nothing but to hope, because nobody can give you any guarantees. . . what I will be able to do later . . . I can hope, and then I can work for it; . . . I can set goals for myself and . . . go as far as possible down that road; . . . I must hope that I can get as far as I really want to get”	(35)
“Just having like confidence and like fearlessness to go and maybe put yourself in situations where you might be a little more vulnerable.”	(27)
**C2. Finding personal meaning**	
Some participants had roles and responsibilities that were meaningful and valuable to them, which motivated them to participate in the community. For example, Fred and Daisy said they had two little dogs that needed walking	(19)
“A few days ago I was lying in my bed, suffering with pain in my legs, I couldn't do anything, just lying there in my bed, doors of my room wide open, but I felt good being home, together when my son came home …”	(38)
Participants stressed that being able to do things just for “killing time” and being able to choose deliberately this kind of ‘occupying time' activity (like listening to the radio or watching television) is very important	(38)
“Everything is still me, I run the household, I am the treasurer of the household, I'm the filer, the mother, I'm the father's voice…”	(27)
**C3. Having a sense of control**	
“I refused the pain medicine, the muscle relaxers, the nerve pain pills … What would have helped through that situation was understanding. Understand that I am not accepting this and do not force me to accept it”	(29)
“People have the right to say what they want to do”	(19)
“I'm going to rehab next week to practice how to get up from the floor and things like that so that I can feel that I am even more independent. This is training that I asked for myself”	(26)
“…she [girlfriend] can do other things for me, and I intentionally had to tell her what she can do and what I want to do”	(39)
**C4. The sense of self**	
“You show them who you are, I tune my car to show people what I can do, some do it in a marginal way, I do it well and people respect me for what I'm doing.”	(40)
Because they “exposed me to everything,” taking him out into the community, Juan was given the opportunity to challenge societal perceptions of disability. He was able to redefine his internal definition of disability, which allowed him to become comfortable to ski, travel alone through Europe, and claim his new identity as a “world traveler”	(27)
“I always had the confidence that no matter what the circumstances were I had value as a person, regardless of being in a wheelchair”	(25)
“It's not the degree of the handicap that decides how your life will be, but rather it's self-image and you really have to work on that. I think many people with spinal cord injuries wrestle with this, especially regarding their male identity.”	(28)
**D. The potential moderators of participation outcome**	
**D1. Gaining knowledge about SCI and oneself**	
Richard relied heavily on the internet to research wheelchair access and equipment: “Absolutely brilliant. I'd be absolutely lost without my best friend Google”	(19)
“Just suss out your own ways of doing stuff, I guess …I think one thing at the spinal unit they sort of tell you this way of doing it and once you get out you realize you can just do it however you want”	(20)
“I remember having the lecture about sex post-spinal injury and it was all about men ... it was quite a long time before anybody told me that I could still get pregnant, or you tend to stop menstruation afterwards ... I didn't even know this was going to happen again”	(21)
“You have to go there [fairs for people with disabilities and service providers] every year. I think everyone with disabilities should do that. Because you have to know what's available”	(22)
**D2. The additive effect of participation**	
“I go out and meet people, I talk to people. […] I'm just there and make something with people I like, meet new people, even in the office here, I meet new people every day”	(9)
Volunteering was also a motivator for social participation. Daisy and Richard were active volunteers and were regularly involved in community life as a result, and they found this very satisfying	(19)
[*Participants*] felt strongly that being out in the community meant that other opportunities for participation would arise	(23)
“Just for guys anyway…if you can do a sport… whether it be darts …or rolling the ball along the ground…or whatever… that's great for getting out there in the community as far as I'm concerned”	(12)
**D3. Behavioral strategies**	
“I'm always preparing, I always ask …is it accessible?”	(24)
“So I made a decision at that time to do everything I could to learn how to do things, and that I would be independent no matter what. So the whole next few months was driven, was based around this goal I set for myself”	(25)
“You try things, taking a trip by a pedal boat, also canoeing. At the rehabilitation center I said I wanted to try canoeing. Of course, I don't have enough hand functions to grasp the paddles. So you have to improvise a bit. Eventually it went well and we canoed a fine 15 kilometers. Well, that was just great”	(22)
Through the support of her friends, Joanna also learned to perform activities in different ways. One example is given by this quote, “Then I said to my friend Sally—I want to visit the solarium, would you like to help me so that I can see if I can make it? You do not have to do anything just stand by my side. Would you do this for me, once, twice or three times so that I feel secure, so that I can do it by myself later? She said, ‘Sure I can do it today without any problem”	(26)
**D4. Human resources**	
“You know when you're first injured you don't really want someone that has not actually been through it to explain to you what's going on and what you're going to go through. . .there is nothing like experiencing it….. Even though I usually tell them the same thing that the therapist has told them. . .it's more reassuring coming from someone who actually lived it”	(27)
“They, the physical therapist and some of the assistant nurses, were really great pillars of support. They listened, they gave suggestions to solve problems, they could explain how others had progressed, they themselves knew what forms it could take, how life could be after you left hospital”	(28)
“I've just about educated all the people I need. I've got a very small group of people I know and work with. Over the years it has all been sorted, basically”	(12)
“I have my best friend. He talks to me all the time, he even cries on the phone with me. He gives me advice, words of wisdom, when I need anything, I can call him and ask him”	(29)
**D5. Communication**	
Elaine [finds it] important “to talk openly from the beginning on, to speak about everything; it quickly becomes a topic and after talking about it people also take you much, yeah, more as a full person, I think”	(30)
“I wasn't open enough with them [health professionals] ... I was very closed about everything—I was quite rude and abrupt at the time, I'd have changed that because it would have helped me”	(21)
“Because I don't know if you asked for help I don't know whether you'd get it. But what I do know is if you don't ask for it, you know, nobody comes and offers”	(31)
“Mostly I make a joke. And if people see that they immediately think: Oh, it's okay”	(22)
**D6. Material resources**	
“[My] family could not chip in, step in, and do the whole care that was needed. So I hired my own attendants and paid for them”	(25)
“It's a world of difference having your own vehicle, just get up and go when you want to”	(32)
“Yeah definitely technology's a huge link to the outside world; having my iPad and then I have my Facebook, therefore I'm linked with people”	(33)
“I need my wheelchair, the loaner is too heavy for me to push”	(34)
**(E). The temporal dimensions of participating in life after SCI**	
**E1. Time course of injury and life**	
“A few months ago I could not even sit up in bed by myself. There is a big difference and I never thought I would get this far. Humans are much stronger that one can imagine, we can climb mountains and I guess soon we will also be able to fly”	(26)
“... and then you really saw for yourself that progress was being made, because sometimes you don't see the small steps forward, but once you've got it on paper that this months I'll learn to sit up in bed by myself, well when you got that, it was like, yes, I've actually done it”	(28)
“Well, the older you get, you can't do everything anymore, right”	(30)
“You're gonna have your downs, but you don't have to stay there”	(25)
**E2. Transition to a new reality**	
“What's going to happen with my life? How do I live in a wheelchair?”	(34)
“I was well prepared but again, it was putting it into practice. You can only practice it so much in [named spinal unit]. You've gotta get out there in the real world to see actually if what you've learnt is practical and we were taught everything as much as we could. The rest was up to us”	(20)
“I think you can prepare people and certainly spending weekends at home and going out and doing lots of things outside the unit is really important—so I think from that point of view you can build it up, but I think there is an element of you having to go back home and getting on with it.”	(21)
“The life I now have to live is about to start, so I cannot just be staying here. We have to try and get started and find out about something; that is to think something up. But at the moment my head is quite empty; I simply don't know what”	(35)
**E3. Adaptation**	
“I just think more people should realize this. Not sticking to what you can't do anymore but looking at what you can do. You'll see that's a lot”	(22)
“I had to find new ways forward, making the changes needed and finding new goals, whether be it finishing a degree or applying for re-training in vocational skills”	(29)
Jake worked to manage others' perceptions of his abilities by “looking the part”, reportedly attending to appearances, and working to demonstrate achieving excellence in valued activities such as work and school	(36)
“I must move on from here where I am now and make the best of it…”	(35)

Although in many countries, progress is being made to improve access to built environments for people with mobility impairments, *the physical environment will always pose challenges* (9, 12, 19, 25, 27–30, 32, 33, 36, 37). There is an acceptance by some that accessibility will always be an issue despite advocacy and policy changes which can reduce barriers. As one participant noted, “In my life, there will never be everything as it should be… so that when you leave the house you do not have to think about anything [barriers], but you can really do the things you want to do” [(25), p. 415].

Individuals face different physical environmental challenges depending on where they live. Footpaths, curb cuts and other infrastructure will vary from one community to the next (19, 29) and the influence of prevailing weather and climatic conditions will vary (23, 32). Sense of physical safety was also mentioned (19, 27). Conversely, environmental challenges that seem to be ubiquitous are those related to transport—including accessible parking and public and private transport services, (26, 29, 32–34) and public bathroom facilities (12, 19, 22, 27, 29).

Across multiple studies, participants reported the need to be proactive to judge whether environmental barriers would be encountered when they ventured to new places (19, 22, 27, 30, 32, 36, 37). One participant described the effect on spontaneity, “…that you almost have to make a terrain assessment beforehand…where am I going, what are the conditions …if only I didn't need to do that” [(32), p. 189]. And being incorrectly advised on accessibility is always a risk (19, 32).

*Society (people) can help and hinder* the participation of people with SCI. As well as physical safety, participants reported a sense of emotional safety in their communities, related to familiarity and a sense of belonging (26, 38). While the support of others in the community is generally welcomed, data suggest that people with SCI would rather ask for help than have others offer it (12, 22, 32, 36). The quality of assistance when rendered may also be a problem (27, 37).

There is understandable sensitivity to what is viewed as condescending or thoughtless behaviors (25, 29, 31, 32). “Yeah, everybody wants to help somebody in a wheelchair,” said one participant [(30), p. 912], or “they're talking over you,” said another [(38), p. 470]. People with SCI describe a desire to be seen as “ordinary” and for the wheelchair to be of no consequence to others in society. “When they look at me I just want them to see me,” said one participant [(38), p. 470].

People with SCI may become worn down by issues of physical access and societal attitudes. This may lead some to constrain their activities, only visiting places that have proved accessible in the past (12, 19, 32), declining social invitations (23), and becoming bound to their own homes (36).

There is also a sense that people with SCI must contest bureaucracies to gain access to formal sources of funding and support. “There are things you have a right to and I think it's wrong to have to fight for them, it's hard enough as it is anyway. It breaks you down,” remarked a participant in relation to obtaining services [(32), p. 188]. The negative effect of “gatekeepers” was noted by participants across a number of studies (19, 25, 28, 32).

### The Personal Physical State

The second analytical theme, (B) *the personal physical state*, comprised two descriptive themes—(B1) *the functional capabilities of an impaired body*, and (B2) *managing health and secondary conditions*. Representative quotes for each theme are presented in Table 2B.

Each individual's degree of impairment and functional capacity will influence their participation choices and the manner in which they participate (12, 20, 23, 29, 30, 33, 36, 38). Being able to transfer independently and/or drive a car are key examples of the direct impact on participation, and achieving certain levels of functional mobility (9, 30).

The perspective that people who sustain incomplete injuries and those who can walk “stand a chance” of achieving participation outcomes is held by some with SCI and doubtless more broadly in society (23). However, altered bladder, bowel, and sexual function (the hidden aspects of SCI) were described by some participants as profoundly impacting participation (12, 19, 22, 23, 29, 30, 36, 37).

Managing the SCI body is, for some, “horribly time consuming” [(27), p. 902] taking time away from participating in life (24). There is also a requirement for vigilance, particularly in relation to skin integrity. One participant stated, “pressure sore…it's at the back of my mind all the time” [(11), p. 7]. The secondary health conditions experienced by people with SCI—skin breakdown, incontinence, fatigue and persistent pain—are a significant impediment to participation (12, 19, 22, 23).

“It's embarrassing for me, but for the people around as well...more so with bowel accidents…but it's the frustration … got your clothes on, you've got into town, [you're] just about to get into the chair and..…you've got to go home,” said a participant [(24), p. 4].

### The Personal Psychological State

The third analytical theme, (C) *the personal psychological state* was evident across four descriptive themes—(C1) *personal feelings, attitudes, and responses* (C2) *finding personal meaning*, (C3) *having a sense of control*, and (C4) *the sense of self* . Representative quotes for each theme are presented in Table 2C.

A great deal of the data found in these articles relates to the theme *personal feelings and attitudes of participants* toward participating in life with SCI. Feelings expressed ranged from embarrassed (19, 23, 31), frustrated (23, 25, 32, 36), and guilty (19, 31, 36, 37), to motivated (12, 19, 22, 25–27, 30, 33, 36), positive (21, 22, 25) and of value to others (25, 27, 35).

Attitudes that encompass flexibility of perspective and the capacity to reframe positively, were part of the psychological context that was linked with positive participation by some participants (22, 25, 27–30, 36, 37). Said one participant, “You have fun in a different way; you find other things, other means, and other ways to have fun” [(32), p. 188]. Closely aligned with flexibility and reframing is the need to frequently weigh up the costs and benefits of acts of participation (12, 23, 24, 27, 32, 36). “Some people have said I should go swimming, said one participant [(11), p. 7].” “But I couldn't be bothered…the time it would take.”

Positive feelings about participation in life are created for some through *finding personal meaning*. Common to some participants across studies was finding meaning through parenting or grandparenting (19, 27–29, 38), undertaking fulfilling employment (9, 12, 25, 36), studying or retraining (26, 29, 36), participating in sport (12, 27), or volunteering (9, 19, 37). For some, meaning was created through participation in activities that were not possible or expected before SCI. “I've been on the Board of Directors [of a housing initiative] for many years. That has been a very, very positive thing, you see. Something else that I never expected to do in my life either is be involved with boards. Like I said, it was probably out of my realm before” [(28), p. 1463].

Some participants expressed that they had found meaning through advocacy and activism (12, 25, 32). But meaning is not always derived in such an overt manner. More subtle ways of finding personal meaning were expressed through creating and maintaining a sense of connectedness and belonging (9, 24, 27, 31, 32, 38). Participants also clearly voiced their decisions NOT to engage in certain roles—employment (19, 23, 37) and sport (12–19, 33, 36, 37) being two examples evident in the data. Choosing to “kill time…[to] clear [the] mind” by completing puzzles or other “non-productive” and solitary pursuits is an important consideration in understanding perceptions of participation in life after SCI [(22), p. 351].

Finding personal meaning is closely linked in this data set with participants *having a sense of personal control* over their participation choices (9, 12, 20, 22, 26, 27, 30, 32, 36, 38, 39). Conversely, many participants expressed the negative impact on their sense of personal control wrought by physical dependency (9, 19, 20, 28, 30, 37), and feelings of insecurity (24, 26, 27, 31, 38). A participant likened this to a return to childhood that is “like you're a little kid again” having to let people know where you are going [(31), p. 1168].

Having a sense of autonomy in the way one participates in life is, to many, the very essence of participation. As one participant explained, “Organizing this barbeque became a part of my life…Some of the neighbors suggested to take over this task,…but I refused,…they can help me, that is no problem, but I will take the lead, just like I did last year and the years before” [(35), p. 5].

The final descriptive theme, grouped within analytical theme C, is *the sense of self* . Participation choices can be a direct result of participants enacting who they are, or who they have become since their SCI (21, 22, 25–28, 30, 36, 38, 39). “I've had jobs that I'm generally proud of …,” said one participant. “Those things have been image enhancing for me [(28), p. 1461].” And another said “You show them who you are, I tune my car to show people what I can do [(35), p. 650].”

Conversely one participant said, “…it was my life if I could fix the roof-gutter or clean the drive-way, this is how I'm conceived…what if I cannot longer…who the hell will I be [(35), p. 7]?” This disconnect between *the sense of self* and the limitation imposed by an impaired body is not impossible to reconcile for some. Participants described some specific examples—engaging with community activities in an organizational rather than physical capacity (25, 39), continuing to enjoy music despite not being able to play the instrument they had previously (38) or substituting new hobbies in place of old hobbies (22). Spirituality and religious beliefs were another form in which participants expressed an ongoing sense of self (22, 25). Being a good friend, someone others can count on, might also remain a constant despite SCI (27, 30).

### The Potential Moderators of Participation Outcomes

The fourth analytical theme, (D) *the potential moderators of participation outcomes*, encompassed six descriptive themes—(D1) *gaining knowledge about SCI and oneself* , (D2) *the additive effect of participation*, (D3) *behavioral strategies*, (D4) *human resources*, (D5) *communication*, and (D6) *material resources*. Representative quotes for each theme are presented in Table 2D.

*Gaining knowledge about SCI and oneself* is a fundamental way in which people with SCI can give themselves the best possible platform for participation in life. This knowledge ranges across learning self-care routines and knowing how to reduce the incidence of secondary conditions (20, 22, 25, 26, 28, 29), knowledge of equipment and technology (12, 19, 22, 32, 33), hobbies and leisure options (12, 22, 25, 33), and understanding how to access sources of support and funding (19, 25, 32). Participants described receiving information and training during rehabilitation programs. Sometimes this was viewed positively and sometimes it was viewed as ill-timed or inadequate (20, 21, 28, 29). Much of the learning described by participants takes place separately from traditional rehabilitation through trial and error (22, 26), learning from peers (12, 21, 22, 25, 27–29, 32, 33, 37), and personal lived experience (22, 27, 32, 34). Rehabilitation was seen by one participant as “…preparing me to [leave] to a basic level” [(20), p. 538].

The second moderator of participation outcomes identified in the data, was *the additive effect of participation* (23). Engaging in sport and recreation (12, 22, 25, 30, 33), volunteering (19, 25, 37, 38), or doing paid work (9, 21, 25), were influential in opening up other opportunities for participants, including social participation (19, 27, 37). When it comes to participation in life, a progressive confidence can arise through trying and succeeding or failing and learning. “I have been thinking about finding some sort of job to start off…hopefully that might lead me to something else somewhere else…,” said one participant [(24), p. 5].

The two descriptive themes, *behavioral strategies*, and *human resources* were the two largest in terms of volume of data derived from the 24 articles. Participants described a number of behaviors they had adopted to improve their participation outcomes, including developing habits and routines (12, 26, 32, 35–37), being organized and planning ahead (19, 22, 24, 27, 28, 33, 36), setting goals and pushing oneself (13, 22, 25–27, 29, 35, 36), and embracing problem solving (12, 19, 22, 35). Exercising assertiveness when necessary (12, 19, 22, 25, 32) and, conversely, using techniques to put others at ease (12, 22, 27, 32) and engaging in reciprocity (12, 22, 26, 27), were examples of behavioral strategies employed when dealing with people.

Some of these behavioral strategies are not without personal cost. “I wish you were free to do what you want without needing to think,” said one participant in relation to the need for constant planning [(32), p. 189].

The impact which *human resources* have on the participation of a person with SCI cannot be overstated. First and possibly foremost is the participation leverage created by friends and family (9, 12, 19, 21, 22, 26–29, 32, 33, 36–38). “After I got out of the hospital they [friends and family] did help a lot. They'd help keep me motivated. Make sure I had things to do. And was not just sitting at home, sitting around,” said one participant [(29), p. 83]. And another remarked,“[Meeting my wife] totally changed my life . . . she's been the biggest impact on my life” [(28), p. 1,464]. But friends and family can also impede the participation of people with SCI. Said one participant, “Sometimes when she [mom] talks to me ... I'm having 14-year-old flash backs where she's trying to tell me what to do, and I'm like I'm 28 years old, a grown man” [(33), p. 1,504].

The camaraderie that may develop with peers was mentioned as important by many participants, and the value of talking with peer mentors who had been through similar experiences was highly valued (12, 19, 21, 22, 25, 27, 28, 31–33, 37). Health professionals could also be vital resources in the life of people with SCI (19, 27, 29, 33). In praise of her occupational therapist, one participant said “She really helped me. She actually took me down to the nursery [workplace] just to say hello to the people and help me feel comfortable” [(37), p. 583].

Unfortunately, some participants expressed the sentiment that they needed more from health professionals or felt let down in their interactions with professionals. Dissatisfaction stemmed from such things as not understanding the person as an individual (20, 21, 28), being treated with a lack of sensitivity and attention to emotional needs (21, 25, 29), or simply that health professionals lacked sufficient (flexibility of) time to spend with participants (21, 28, 31, 35).

Whether it be family and friends, peers, professionals, or organizations, participants saw value in having allies to “navigate” the challenges encountered when attempting to participate in life (34). As one participant stated, “…having somebody to at least guide you or show you ‘this is the way to go', not just a little binder that says how to do your bowels, how to go to the bathroom and watch out for pressure sores … what I am talking about is every day the more complex solutions that would [have] allowed us to figure things out…” [(36), p. 4].

*Communication*, the fifth descriptive theme within the potential moderators, is a life skill that takes on added importance for people with SCI. Participants recognized a benefit in “openness” in their communication style (12, 21, 26, 27, 30, 31), and displayed examples of the benefits of negotiation skills (27) and use of humor (12, 19, 22). For one participant, to feel safe and confident he needed to communicate explicitly with his partner and neighbors. “I wanted to know if my wife would take care of me, I wanted to know if the next-door neighbors could come over once in a while and see if I am alright that kind of stuff, can you see otherwise I would not feel safe” [(22), p. 352].

The final moderator evident in the data was *material resources*. Access to personal care support (19, 20, 23, 32), equipment and technology (12, 19, 23, 25, 29, 32–34), and transport (9, 23, 26, 29, 30, 32–34) were integral to participants' successful participation. “I get this electric wheelchair, suddenly I can go down the hallway and I can visit anybody ...,” is a simple statement that encapsulates the power of having the material resources required to participate [(30), p. 910]. Vital to gaining these material resources are adequate sources of funding, provided in a transparent and timely way (12, 19, 24, 25, 29, 30, 32–34, 36). In addition, specialized professional support helps people with SCI through the process of acquiring resources (19, 27, 33).

### The Temporal Dimensions of Participating in Life After SCI

The fifth analytical theme, (E) *the temporal dimensions of participating in life after SCI*, evolved from three descriptive themes—(E1) *the time course of injury and life*, (E2) *the transition to a new reality*, and (E3) *adaptation*. Representative quotes for each theme are presented in Table 2E.

Participation will differ for people with SCI depending on where they are in *the time course of injury and life* (12, 20, 21, 23, 25–29, 33, 37). For example, a newly injured individual may have a relatively constrained view of their potential to participate in life after SCI. People will not have a full understanding of their participation possibilities. This sentiment was summed up by one participant. “The life I now have to live is about to start, so I cannot just be staying here. We have to try and get started and find out about something; that is to think something up. But . . . at the moment my head is quite empty; I simply don't know what” [(21), p.45].

“People aren't static [(11), p. 4].” People have ups and downs in life (12, 25). Their aspirations, abilities and circumstances will change throughout life. Age at time of injury, as well as the process of aging, are also factors which will influence participation choices and outcomes (30).

The descriptive theme, *transition to a new reality* speaks to both emotional and pragmatic processes that participants described as they are faced with the reality of SCI (20, 21, 25, 27–29, 34). Leaving a rehabilitation setting to confront the real world (12, 20), and leaving behind the physical and emotional safety (20, 31), speak to a very specific time of transition that is a challenging experience for many. One participant put it thus, “I just wanted to go back in hospital honestly and for somebody who was so keen to get out, I wanted to get back to my support group [(38), p. 467].”

The final descriptive theme, *adaptation* is a construct that speaks to mind shifts undertaken by people with SCI. Acknowledging change (21, 22, 25, 28, 32, 39, 40), displaying degrees of flexibility in choice making (12, 19, 25, 40) and “find new ways forward” [(36), p. 5] embody this theme.

## Discussion

The aim of this thematic synthesis of qualitative research findings was to deepen understanding of the influences on people's participation in life after spinal cord injury. Using a process of thematic analysis and synthesis, the participant quotes drawn from 24 studies, comprising a total of 352 people with SCI from 12 countries, produced five analytical themes which encompass 17 descriptive themes. Considered together, we describe these analytical themes as the *interconnected elements that influence participation after SCI*.

The results of the thematic synthesis show the ubiquitous influences of the physical environment. Similarly, and unsurprising, is the evidence for the influence of the social environment, along with the health and physical function of individuals with SCI. These influences have featured prominently in much of the observational, quantitative research that has been conducted in this area (41–45).

Perhaps more important for advancing our understanding of the influences on participation, are the number of themes identified that relate to the individual's psychological state. Understanding the individual is acknowledged as important for providing person-centered care after SCI (46). However, extending this understanding of the individual to their inner psychological world—personal attitudes, personal meanings, how they understand control and autonomy, their sense of self and their inner resources, may be far deeper than most practitioners and support personnel would deliberately delve.

Psychotherapeutic interventions may be offered in some instances, principally to augment personal coping (47), however there may be far more scope for addressing each person's internal psychological context through positive psychological programs (48, 49). The interactive nature of personal meaning, sense of control and sense of self is, without doubt, extremely complex. Nonetheless, it may only be through first considering each construct individually that more holistic therapeutic interventions can arise.

The potential moderators of participation outcomes form a substantial portion of the findings of this review. All the moderators described are, to some degree, modifiable. Gaining the knowledge to manage one's body after SCI, and learning, practicing and using behavioral strategies, such as use of assertiveness, habit formation, pre-planning and socially positive communication, may be trainable, at least to some degree, for all with SCI. Involvement in activities to expand participation options (particularly social participation) is something that people with SCI can be supported to undertake—as long as it is individualized to their interests. There have been but a handful of programs touching on these aspects that have been evaluated and reported in the literature (50–55).

Participation outcomes after SCI are not just about what a person knows but who they know. People who sustain an SCI may or may not have a strong network of human resources at the time of injury. We know that some networks will break down while others will not. It would appear that those with robust networks may have a participation head start (56). When this is not the case, proactive measures to aid people to develop their networks of support are important (57).

Optimizing the material resources of equipment, technology, transport, and care support starts with (uncomplicated) access to appropriate levels of funding. However, getting the optimal material resources also rests on much more than just money. Information about, and access to, the latest equipment and technology, as well as professional support to make informed choices is critical (58–60). Similarly, the quality of support staff and their training will always be crucial for people with SCI to leverage the best participation outcomes.

Time is a crucial variable in participation outcomes. The temporal dimensions of participating in life after SCI have been highlighted in previous work on undergoing transitions (61–63), trajectories of outcomes (64, 65), and aging with SCI (66, 67).

Adaptation is the final temporal descriptive theme. The term *psychosocial adjustment* is the term commonly used in the past which is similar to adaptation (68). However, *adaptation* may be considered more holistic—encompassing physical adaptations, behavioral adaptations, shifts in mind set and post traumatic growth, all of which may be called on as each person with SCI finds their path through life.

### Findings in Relation to Biopsychosocial Models Such as the ICF

It has not been the purpose of this synthesis to replace existing frameworks of understanding but to provide alternate ways through which to view the influences on participation for those with SCI. The results of this synthesis clearly affirm the biopsychosocial foundations of the lived experience of SCI. Though it was not the purpose of this review to use the ICF as a framework for thematic categorization, it is abundantly clear that the results would map strongly to its components. The *external contextual influences* correspond with the Environment component (physical and societal domains) of the ICF. The *personal physical state* closely aligns with the ICF components Body Structure and Function, Activities and Participation. The *personal psychological state* has some overlap with the Mental Functions described in Body Structure and Function but might also overlap with Personal Factors.

The *potential moderators of participation outcomes* would appear to cross bio, psycho and social elements of influence. *Gaining knowledge about SCI and oneself* are aspects of managing biological functions. Leveraging the *additive effects of participation*, and employing *behavioral strategies* are psychological tools for changing participation outcomes, and, access to *human and material resources* and *communicating effectively* are social moderators.

The *temporal dimension of participating in life after SCI* brings an aspect which is difficult to show in a framework such as the ICF. Classifications are recorded at snapshots in time, so a temporal dimension can be captured by repeating the classification process over time.

### Implications for Service Provision

It goes without saying that assisting people to optimize their physical capacity will remain a mainstay of rehabilitation. This includes maintaining health and wellness and minimizing secondary complications of SCI. However, the continuing predominance of physical function interventions as the mainstay of rehabilitation therapies for people with SCI, coupled with the medical focus on symptom and bodily system based care (31), should perhaps be questioned.

Understanding the personal psychological context is something that is essential to person centered rehabilitation. It isn't just a matter of intervention when pathological states are diagnosed. It is about positively emphasizing existing psychological strengths and giving people opportunity to develop their capacities where challenges are identified.

There is a great deal of scope in rehabilitation programs for examining moderators and determining if they can be shaped through various interventions. Can the behavioral strategies demonstrated in this data be taught to people with SCI?

Friends and family as moderators of participation outcomes must be an integral part of the rehabilitation process. The power of peer led programs needs to be assessed and refined (69). Bringing together the expertise and perspectives of practitioners and peers may be the best way to optimize knowledge and information provision for people with SCI.

Rehabilitation institutions still tend to operate on models that are heavily weighted toward a single episode of primary rehabilitation (70). Service providers must recognize the evolving nature of each person's personal journey with SCI. Things change with time and services must be more fluid and less fixed. There is no fixed timetable for adaptation to SCI and people with SCI will experience episodic need for support (71). To provide contemporary person-centered rehabilitation services, we need to understand participation in life after SCI as a function of time.

### Review Limitations

It is always difficult to put boundaries around the construct of participation. Consequently, the mapping and targeting of articles for this review has been very iterative in nature. Although this is consistent with a hermeneutic approach to literature review (72, 73) bias may be at play in decisions to include or reject certain lines of enquiry. By being as open as possible about each of the iterations, it is hoped that others can judge the merits of the decision making.

Narrowing the context to certain countries with highly developed economies and “western cultures” may be questionable as a strategy for creating homogeneity. The reality is these countries may be comprised of many multicultural communities, First Nations communities and rural and remote communities, all of which may create heterogeneity of experience for people with SCI. In addition, the funding and support structures for people with disability will be very different from one country to the next. As with all qualitative research, each reader must view the results with a degree of caution and reflect on the transferability of findings to differing contexts.

The decision to focus on quotes and paraphrases included in the final manuscript of primary articles may be contentious. Typically, in meta ethnography or qualitative meta synthesis, the entirety of the interpretations and conclusions in the articles would be considered as data (74). However, our interest was to hear the voice of the people with SCI which comes to the fore in these articles. Approaching the synthesis of these articles in a different manner may yield different insights.

We acknowledge that this synthesis relies on data which is weighted toward the early months and years after SCI. The lived experience of rehabilitation and transition to life in the community have understandably been a strong focus of scholarship, which may be compounded by a tendency for participants to re-explore this pivotal time in their lives when asked to describe their lived experiences, despite being many years post injury. While several longitudinal quantitative studies have examined long term outcomes, an important step forward from the current study would be deeper qualitative investigation of the *life course* experience of SCI.

We believe that to some extent the limitations of the current review are mitigated by the unique approach adopted. Having an overview of the views of 352 participants with SCI across 12 countries, and from a range of study contexts, has enabled us to gain valuable insights into key aspects of participation after SCI.

### Next Steps

Our understanding of these *interconnected elements that influence participation after SCI* can be nuanced by examining the literature which is specific to particular participation constructs (e.g., employment, recreation etc). We can also learn more by examining the mechanism by which specific influences create participation outcomes (e.g., secondary conditions, peer mentoring).

The broad range and the relative priorities of factors identified in the current review provide a potential guide for future funding models and policy statements. In order to meaningfully boost the participation of people with SCI, policy settings and funding equations must recognize the breadth of factors involved and the relative importance of some factors (e.g., psychosocial wellbeing) over others. Most importantly, there is a need for development, evaluation and dissemination of programs that seek to harness these influences on participation.

## Conclusion

Synthesis of qualitative inquiry provides valuable insights into the perceptions of influences on participation in life from the point of view of people with SCI. It provides a greater depth than relying on the ICF alone to understand participation. From this synthesis, we surmise that participation outcomes may be enhanced by greater emphasis in rehabilitation settings on understanding a person's psychological state. There also may be capacity to leverage the moderators of participation through specific interventions and programs to improve participation outcomes.

## Author Contributions

DA and SS performed the database searches, article selection, and data extraction. DA performed the primary synthesis and created the first draft of this article. MK and PK validated the synthesis. SS, MK, and PK provided significant editing. All authors contributed to the design of this review, contributed to the article, and approved the submitted version.

## Funding

Funding for open access publication of this manuscript was provided by the Metro South Health—Study, Education, and Research Trust Account (SERTA).

## Conflict of Interest

The authors declare that the research was conducted in the absence of any commercial or financial relationships that could be construed as a potential conflict of interest.

## Publisher's Note

All claims expressed in this article are solely those of the authors and do not necessarily represent those of their affiliated organizations, or those of the publisher, the editors and the reviewers. Any product that may be evaluated in this article, or claim that may be made by its manufacturer, is not guaranteed or endorsed by the publisher.
