# Supplementary material for: Influences on Participation in Life After Spinal Cord Injury: Qualitative Inquiry Reveals Interaction of Context and Moderators
Source: Front Rehabil Sci. 2022 May 31;3:898143. doi: 10.3389/fresc.2022.898143 (PMC9397943; doi:10.3389/fresc.2022.898143)
Supplement: Supplementary file 1 [file Table_1.DOCX]

**Supplementary material 1: Search Strings**

**Research question:** *What do people with SCI perceive to be the influences on their participation in life*?

**Search structure:** SPIDER Sample (people with SCI), Phenomenon of Interest (Participation), Design (interview, etc) Evaluation (influences), Research Type (qualitative)

*Design* and *Research Type* have been combined for search purposes.

**CINAHL & MEDLINE via EBSCOhost**

((TI ( spinal cord injury or sci or paraplegia or quadriplegia or tetraplegia ) OR AB ( spinal cord injury or sci or paraplegia or quadriplegia or tetraplegia ))

AND

(TI ( community integration or social inclusion or community reintegration or participat* ) OR AB ( community integration or social inclusion or community reintegration or participat* ))

AND

(TI ( influence* OR barrier* OR facilitat* OR determin* OR associate* OR predict* OR relationship* OR perception* OR perceive* OR experience* OR strateg* ) OR AB ( influence* OR barrier* OR facilitat* OR determin* OR associate* OR predict* OR relationship* OR perception* OR perceive* OR experience* OR strateg* ))

AND

(TI ( qualitative OR interview* OR focus group* OR narrative* OR photo* OR video* OR diary OR story OR case stud* OR constant comparison OR content analysis OR conversation* OR discourse OR ethno* OR grounded theory OR hermeneutic OR phenomenology* OR thematic OR theme*) OR AB ( qualitative OR interview* OR focus group* OR narrative* OR photo* OR video* OR diary OR story OR case stud* OR constant comparison OR content analysis OR conversation* OR discourse OR ethno* OR grounded theory OR hermeneutic OR phenomenology* OR thematic OR theme*)))

Limiters: Peer reviewed, English language, Publication date range Jan 2006 - Dec 2021

**PsycInfo via Ovid**

((( spinal cord injury OR sci OR paraplegia OR quadriplegia OR tetraplegia ) TI OR

( spinal cord injury OR sci OR paraplegia OR quadriplegia OR tetraplegia ) AB )

AND

(( community integration OR social inclusion OR community reintegration OR participat* ) TI

OR ( spinal cord injury OR sci OR paraplegia OR quadriplegia OR tetraplegia ) AB )

AND

(( barrier* OR facilitat* OR determin* OR associate* OR predict* OR relationship* perception* OR perceive* OR experience* OR strateg* ) TI OR ( barrier* OR facilitat* OR determin* OR associate* OR predict* OR relationship* OR perception* OR perceive* OR experience* OR strateg* ))

AND

(( qualitative OR interview* OR focus group* OR narrative* OR photo* OR video* OR diary OR story OR case stud* OR constant comparison OR content analysis OR conversation* OR discourse OR ethno* OR grounded theory OR hermeneutic OR phenomenology* OR thematic OR theme*) TI OR ( qualitative OR interview* OR focus group* OR narrative* OR photo* OR video* OR diary OR story OR case stud* OR constant comparison OR content analysis OR conversation* OR discourse OR ethno* OR grounded theory OR hermeneutic OR phenomenology* OR thematic OR theme*) AB ))

Limiters: Peer reviewed journal, English language, Publication date range Jan 2006 - Dec 2021

CINAHL

http://libraryproxy.griffith.edu.au/login?url=https://search.ebscohost.com/login.aspx?direct=true&db=ccm&bquery=((TI+(+spinal+cord+injury+or+sci+or+paraplegia+or+quadriplegia+or+tetraplegia+)+OR+AB+(+spinal+cord+injury+or+sci+or+paraplegia+or+quadriplegia+or+tetraplegia+))+AND++(TI+(+community+integration+or+social+inclusion+or+community+reintegration+or+participat*+)+OR+AB+(+community+integration+or+social+inclusion+or+community+reintegration+or+participat*+))+AND++(TI+(+influence*+OR+barrier*+OR+facilitat*+OR+determin*+OR+associate*+OR+predict*+OR+relationship*+OR+perception*+OR+perceive*+OR+experience*+OR+strateg*+)+OR+AB+(+influence*+OR+barrier*+OR+facilitat*+OR+determin*+OR+associate*+OR+predict*+OR+relationship*+OR+perception*+OR+perceive*+OR+experience*+OR+strateg*+))+AND+(TI+(+qualitative+OR+interview*+OR+focus+group*+OR+narrative*+OR+photo*+OR+video*+OR+diary+OR+story+OR+case+stud*+OR+constant+comparison+OR+content+analysis+OR+conversation*+OR+discourse+OR+ethno*+OR+grounded+theory+OR+hermeneutic+OR+phenomenology*+OR+thematic+OR+theme*)+OR+AB+(+qualitative+OR+interview*+OR+focus+group*+OR+narrative*+OR+photo*+OR+video*+OR+diary+OR+story+OR+case+stud*+OR+constant+comparison+OR+content+analysis+OR+conversation*+OR+discourse+OR+ethno*+OR+grounded+theory+OR+hermeneutic+OR+phenomenology*+OR+thematic+OR+theme*)))&cli0=DT1&clv0=200601-202212&type=1&searchMode=Standard&site=ehost-live&scope=site

[386]

MEDLINE

http://libraryproxy.griffith.edu.au/login?url=https://search.ebscohost.com/login.aspx?direct=true&db=cmedm&bquery=((TI+(+spinal+cord+injury+or+sci+or+paraplegia+or+quadriplegia+or+tetraplegia+)+OR+AB+(+spinal+cord+injury+or+sci+or+paraplegia+or+quadriplegia+or+tetraplegia+))+AND++(TI+(+community+integration+or+social+inclusion+or+community+reintegration+or+participat*+)+OR+AB+(+community+integration+or+social+inclusion+or+community+reintegration+or+participat*+))+AND++(TI+(+influence*+OR+barrier*+OR+facilitat*+OR+determin*+OR+associate*+OR+predict*+OR+relationship*+OR+perception*+OR+perceive*+OR+experience*+OR+strateg*+)+OR+AB+(+influence*+OR+barrier*+OR+facilitat*+OR+determin*+OR+associate*+OR+predict*+OR+relationship*+OR+perception*+OR+perceive*+OR+experience*+OR+strateg*+))+AND+(TI+(+qualitative+OR+interview*+OR+focus+group*+OR+narrative*+OR+photo*+OR+video*+OR+diary+OR+story+OR+case+stud*+OR+constant+comparison+OR+content+analysis+OR+conversation*+OR+discourse+OR+ethno*+OR+grounded+theory+OR+hermeneutic+OR+phenomenology*+OR+thematic+OR+theme*)+OR+AB+(+qualitative+OR+interview*+OR+focus+group*+OR+narrative*+OR+photo*+OR+video*+OR+diary+OR+story+OR+case+stud*+OR+constant+comparison+OR+content+analysis+OR+conversation*+OR+discourse+OR+ethno*+OR+grounded+theory+OR+hermeneutic+OR+phenomenology*+OR+thematic+OR+theme*)))&cli0=RV&clv0=Y&cli1=DT1&clv1=200601-202212&type=1&searchMode=Standard&site=ehost-live&scope=site

[421]

PsychINFO

https://libraryproxy.griffith.edu.au/login?url=https://ovidsp.ovid.com/ovidweb.cgi?T=JS&NEWS=N&PAGE=main&SHAREDSEARCHID=nf8hTUiXNDVI8z7iKBUvvhgnHsdaJ7L7VaDRUjk4ep948iwddbtFlPPPgQKeb2CG

[181]
