# Supplementary material for: Influences on Participation in Life After Spinal Cord Injury: Qualitative Inquiry Reveals Interaction of Context and Moderators
Source: Front Rehabil Sci. 2022 May 31;3:898143. doi: 10.3389/fresc.2022.898143 (PMC9397943; doi:10.3389/fresc.2022.898143)
Supplement: Supplementary file 2 [file Table_2.DOCX]

S2: Attributes of articles investigating influences on participation in a general/exploratory way

| **First Author** | **Year** | **Title** | **Country study conducted** | **Purpose of study** | **Participants** | **Data collection methods** | **Data analysis** |
| --- | --- | --- | --- | --- | --- | --- | --- |
| Amsters | 2018 | Determinants of participating in life after spinal cord injury - advice for health professionals arising from an examination of shared narratives | Australia | A research project was developed based on a person-perceived approach, to examine the topic of determinants of participating in life after SCI. | 4 people with SCI (2 female, 2 male) with significant lived experience and opinions to share; aged 39 to 52; time since injury 17 to 31 years | Focus group; unstructured interviews | Thematic analysis |
| Angel | 2009 | Getting on with life following a spinal cord injury: regaining meaning through six phases | Denmark | The purpose of the present study, therefore, was to investigate the interpretive processes that patients with spinal cord injury move through to regain meaning in their new life situation. | 12 people with traumatic SCI (6 female, 6 male); aged 18-73; in the first 2 years after injury | Narrative interviews and field observations | Three level textual analysis: naïve reading, structural analysis, critical interpretation |
| Barclay | 2015 | Facilitators and barriers to social and community participation following spinal cord injury | Australia | This paper reports part of a larger study that aimed to explore the experience and meaning of social and community participation of people living with an acquired SCI in Victoria, Australia. | 17 community dwelling people with SCI (4 female, 13 male); aged 18-85 years; time since injury 4-29 years | Semi structured interviews | Thematic analysis |
| Barclay | 2017 | Understanding the factors that influence social and community participation as perceived by people with non-traumatic spinal cord injury | Australia | The objective of this paper is to present the experiences of social and community participation of adults living with NTSCI, and the factors that influence that participation, and to discuss strategies that occupational therapists can use when working with their NTSCI clients. | 17 community dwelling people with non-traumatic SCI (8 female, 9 male); aged 33-83 years; time since injury less than 1 year to 61 years | Semi structured interviews | Inductive thematic analysis using constant comparative approach |
| Barclay | 2019 | The experiences of social and community participation of people with non-traumatic spinal cord injury | Australia | The aim for this part of the study was to explore the experience of returning to social and community participation following non-traumatic SCI | 17 community dwelling people with non-traumatic SCI (8 female, 9 male) aged between 18-85 years; time since injury from less than 2 years to more than 10 years | Semi structured interviews | Inductive thematic analysis |
| Bartolac | 2019 | Understanding the everyday experience of persons with physical disabilities: building a model of social and occupational participation | Croatia | We posed two research questions: 1. How do persons with physical disabilities describe their experience of occupational and social participation? 2. Do persons with physical disabilities experience occupational deprivation and social marginalization and, if yes, how do they describe this experience? | 15 adults with neurological physical disabilities (9 females, 6 males), age range 20 to 65.  Three participants had SCI, time since injury not specified | Semi structured interviews; focus group | Constant comparative method |
| Carr | 2017 | Community participation for individuals with spinal cord injury living in Queensland, Australia | Australia | The aim of the current study was to determine why some individuals with SCI, who live in Queensland, Australia, regularly participate in the community while others do not. | Qualitative phase: Separate sample of 8 people with SCI (1 female, 7 male); aged 31 to 62; time since injury 1 to 29 years | Mixed methods; semi structured interviews; focus group | Grouping of data into themes determined in quantitative phase |
| Dickson | 2011 | Difficulties adjusting to post-discharge life following a spinal cord injury: an interpretative phenomenological analysis | UK | The aim of our research was to capture the lived experience of SCI from the perspective of the individuals who live with it. | 17 people with SCI (3 female, 14 male); aged 26 to 62; C5, C6 or C7 tetraplegia; time since injury 17 months to 32 years | Unstructured interviews | Interpretive phenomenological analysis |
| Fritz | 2015 | Long-term community reintegration: concepts, outcomes and dilemmas in the case of a military service member with a spinal cord injury | USA | The purpose of this paper is to characterize long-term outcome-related resources and barriers to meaningful community participation. | A 28 year old male military veteran with service-related traumatic SCI, 4 years post injury | Semi structured interviews | Content analysis |
| Hall | 2021 | Perspectives on life following a traumatic spinal cord injury | USA | This study sought to explore the lived experiences of individuals with SCI after their injury, as well as obtain the perspectives of therapists working with SCI patients | 10 people with traumatic SCI (1 female, 9 male); aged 29 to 65 years; time since injury 1 to 20+ years | Semi structured interviews | Grounded theory Thematic analysis |
| Isaksson | 2010 | One woman's story about her everyday life after a spinal cord injury | Sweden | The aim was to describe and offer an explanation for how one woman viewed her everyday life after a spinal cord injury. | 1 woman with SCI aged 25 at injury; purposeful sampling from an earlier study based on the richness of information in her stories; time since injury 1 year at initial interviews and 2 years at final interview | Semi structured interview | Narrative analysis |
| Nunnerley | 2013 | Leaving a spinal unit and returning to the wider community: an interpretative phenomenological analysis | New Zealand | The purpose was to understand the lived experience of the transition from a specialist SCI rehabilitation setting to participation in the community through listening to the accounts of people with SCI | 9 people (2 female, 7 male) with traumatic SCI within 12 months of discharge from a specialist spinal injury unit; age range 20 to 55. | Semi structured interviews | Interpretive Phenomenological Analysis (IPA) |
| Price | 2011 | Beyond my front door: the occupational and social participation of adults with spinal cord injury | USA | This article presents analysis of an ongoing study that examines the life satisfaction and occupational and social participation of individuals living in the community 1 to 5 years after spinal cord injury. | 11 community dwelling people, male and female; 1-5 years post SCI; age not specified | Semi structured interviews | Inductive narrative analysis |
| Reinhardt | 2013 | "It takes two to tango" revisited: a qualitative study on integration and participation of people living with spinal cord injury in Switzerland | Switzerland | We aimed to examine how integration and participation are understood by persons with SCI. | 14 (9 female, 5 male) community dwelling adults living with SCI for 3 to 49 years; 1 person had multiple sclerosis, 2 had spina bifida. | Semi structured interviews | Iterative coding based on grounded theory |
| Ripat | 2012 | Self-perceived participation among adults with spinal cord injury: a grounded theory study | Canada | The purpose of this study was to advance an understanding of how adults with SCI participate in their daily life and within their communities, to promote opportunity for participation. | 19 people (6 female, 13 male) with SCI; age range 20 to 60+; time since injury not specified | Semi structured interviews; focus group; photovoice | Constant comparative analysis |
| Ruoranen | 2015 | Participation and integration from the perspective of persons with spinal cord injury from five European countries | Finland, Germany, Ireland, Northern Ireland (UK), Switzerland | This research was designed to compare the subjective understanding of participation and integration among persons with SCI from 5 European countries. | 54 people with acquired SCI and 3 people with spina bifida (18 female, 39 male); age range 20 to 75; time since injury 3 to 46 years (spina bifida excluded) | Semi structured interviews | Qualitative content analysis |
| Sand | 2006 | Spinal cord injured persons' conceptions of hospital care, rehabilitation, and a new life situation | Sweden | The purpose of this study was to describe how persons with SCI experience their rehabilitation process in order to identify areas that need to be improved and suggest avenues for change. | 19 people (3 female, 16 male) with complete C5 C6 or C7 SCI; aged 19 to 64; 2 to 10 years post injury | Semi structured interviews | Modified descriptive version of phenomenographic analysis |
| Silver | 2012 | Barriers for individuals with spinal cord injury returning to the community: a preliminary classification | USA | The purpose of this project was to identify the barriers encountered by individuals with acute SCI as they attempt to reintegrate into their community during the challenging first year post inpatient rehabilitation. | 26 people (5 female, 21 male) with SCI; aged 18 to 86; in the first year after discharge from inpatient rehabilitation (and having been admitted to inpatient rehabilitation within a year of injury) | Mixed methods; open questions as part of survey | Categorisation of barriers |
| Suddick | 2009 | Reintegration and rehabilitation after spinal cord injury: a small-scale pilot study | UK | The purpose of this pilot study was to explore the lived experience of community reintegration for a small group of people living with SCI in the UK and how rehabilitation was perceived to have helped or hindered this reintegration. | 7 people (4 female, 3 male) with SCI, aged 24 to 38; 5-12 years post SCI and undergone some form of rehabilitation | Semi structured interviews | Adapted thematic content analysis |
| Van de Velde | 2010 | Perceived participation, experiences from persons with spinal cord injury in their transition period from hospital to home | Belgium | It is the aim of this study to add to the existing body of knowledge of participation by exploring the 'person-perceived participation' in individuals with SCI. | 11 males with SCI who were in their transition from hospital to home (2 months post discharge); all with paraplegia, employed before injury, living with a partner; aged 25 to 56 | Semi structured interviews | Constant comparative method |
| van de Velde | 2012 | The illusion and the paradox of being autonomous, experiences from persons with spinal cord injury in their transition period from hospital to home | Belgium | The research question is ‘how do individuals with SCI experience autonomy in their transition period from the rehabilitation hospital to home? | 11 males with SCI; aged 25-56 years; in their transition from hospital to home (2 months post discharge); all with paraplegia, employed before injury, living with a partner | Semi structured interviews | Constant comparative method |
| van de Velde | 2013 | How do men with paraplegia choose activities in the light of striving for optimal participation? A qualitative study, based on a phenomenological hermeneutical method | Belgium | It remains unclear how individuals with SCI choose their activities and what happens during this process. It was the goal of this study to focus on this choice-making process as it is experienced because this aspect remains under-investigated. | 12 males with thoracic level SCI; aged 25 to 56 years; in their transition from hospital to home (up to 2 months post discharge) and employed/ earning a living before injury | Semi structured interviews | Structural thematic analysis |
| van de Ven | 2008 | Strategies for autonomy used by people with cervical spinal cord injury: a qualitative study | Netherlands | Our aim was to explore the experiences of people with cervical SCI as regards autonomy, in order to identify successful strategies. | 8 people (3 female, 5 male) with C4 to C7 level SCI and members of quad rugby clubs; aged 27 to 55; mean time since injury 14 years | Semi structured interviews | Selected passages coded, thematically described and categorised based on dimensions of autonomy |
| Weitzner | 2011 | Getting on with life: positive experiences of living with a spinal cord injury | Canada | The purpose of this study was to identify how individuals living with SCI viewed and/or used their disabilities positively. | 52 community dwelling people with SCI (12 female, 40 male); current age not specified; time since initial rehabilitation 3 to 17+ years | Semi structured interviews | Secondary analysis of data using a grounded theory approach |
