# Supplementary material for: Influences on Participation in Life After Spinal Cord Injury: Qualitative Inquiry Reveals Interaction of Context and Moderators
Source: Front Rehabil Sci. 2022 May 31;3:898143. doi: 10.3389/fresc.2022.898143 (PMC9397943; doi:10.3389/fresc.2022.898143)
Supplement: Supplementary file 3 [file Table_3.DOCX]

S3 Attributes of articles investigating focussed/specific influences on participation - not included in synthesis

| **First Author** | **Year** | **Title** | **Country study conducted** | **Purpose of study** | **Sample demographics** | **Data collection methods** | **Data analysis** | **Influence in focus** |
| --- | --- | --- | --- | --- | --- | --- | --- | --- |
| Beauchamp | 2016 | Peer mentoring of adults with spinal cord injury: a transformational leadership perspective | Canada | The overall purpose of this study was to examine the peer mentoring experiences of adults with a SCI, and the extent to which the peer mentoring behaviours used by mentors align with transformational leadership behaviours | 15 mentees with SCI (7 female, 8 male); aged 25-69 | Semi structured interviews | Content analysis | Peer mentoring |
| Bourke | 2019 | Using cannabis for pain management after spinal cord injury: a qualitative study | New Zealand | The aim of this study was to understand why individuals with SCI choose to use cannabis to manage their pain and their experiences of doing so. | 8 people with SCI (2 female, 6 male) who use cannabis for pain | Semi structured interviews | Thematic analysis | Pain |
| Boyce | 2012 | Active recreation and well-being: the reconstruction of the self identity of women with spinal cord injury | USA | The purpose of this study was to increase awareness of and knowledge about active recreation and well-being for women with disabilities by acquiring the perspectives of female athletes who had incurred a SCI. | 3 women with adult-onset SCI and 1 woman with spina bifida; participating in adaptive sports programs | Semi structured interviews | Constant comparison | Active recreation |
| Burns | 2015 | Phenomenological study of neurogenic bowel from the perspective of individuals living with spinal cord injury | Canada | This study used a phenomenologic (qualitative) design to investigate the experience of living with NBD (neurogenic bowel dysfunction) after an SCI. | 19 people with SCI (13 male, 6 female) | Semi structured interviews | Phenomenologic analysis - typologies, deep descriptive methodology | Neurogenic bowel dysfunction |
| Callaway | 2015 | Secondary health conditions experienced by people with spinal cord injury within community living: implications for a national disability insurance scheme | Australia | The aim of the current study was to determine why some individuals with SCI, who live in Queensland, Australia, regularly participate in the community while others do not. | 33 people with SCI (11 female, 22 male); average age 58.5; average time since injury 20 years | Mixed methods; semi structured interviews | Thematic analysis | Secondary health conditions |
| Carver | 2016 | The impact of mobility assistive technology devices on participation for individuals with disabilities | USA | The purpose of this study is to understand how Mobility Assistive Technology Devices (MADTs) impact participation for individuals with disabilities. The authors aim to address the gap in research and contribute to the body of knowledge on the perspectives AT device users have toward their MATDs. | 61 users of mobility assistive technology devices; aged over 18 years; the number of people with SCI is not specified | Mixed methods; open questions as part of survey | Common themes were identified | Mobility assistive technology devices |
| Chemtob | 2018 | Exploring the peer mentorship experiences of adults with spinal cord injury | Canada | The objective of this qualitative study was to understand SCI peer mentorship through a self-determination theory lens by gathering mentees’ perceptions of their experiences with their mentors. | 13 people with SCI (4 female, 9 male) who had been involved in at least 4 peer mentorship interactions over the past 5 years; aged over 18 years | Semi structured interviews | Inductive and deductive content analysis | Peer mentorship experiences |
| Cote-Leclerc | 2017 | How does playing adapted sports affect quality of life of people with mobility limitations? Results from a mixed-method sequential explanatory study | Canada | The objective of this study was to: 1) compare the subjective quality of life of adults with mobility limitations playing a wheelchair adapted sport to that of a population reporting no mobility limitations, and 2) explore the influence of playing an adapted sport on the quality of life of adults with mobility limitations. | 34 manual wheelchair users who played wheelchair adapted sports; aged from 18 to 62; 10 of these participated in the qualitative phase (5 female, 5 male); 8 of these had paraplegia or tetraplegia. | Mixed methods; semi structured interviews | Thematic content analysis | Adapted sports |
| Ekelman | 2017 | A wellness program for men with spinal cord injury: participation and meaning | USA | The purpose of this study is to explore how men with SCI describe their experiences and meaning of participating in a wellness centre program, how they perceived these experiences as influencing their well-being, and how these experiences relate to co-occupations and occupational spin-off concepts. | 4 men with SCI aged 26-49 who were members of a specific community wellness centre | Interviews and observations | Thematic analysis | Wellness program |
| Folan | 2015 | Exploring the experience of clients with tetraplegia utilizing assistive technology for computer access | Australia | The aim of this pilot study was to gain an understanding of the experiences of clients with tetraplegia trialling assistive technologies for computer access during different stages in a public rehabilitation service. | 7 people with SCI aged 20 to 60; tetraplegia with significant upper limb impairment; post-acute but recent injury; exposure to assistive technology for computer access | Semi structured interviews | Thematic analysis | Assistive technology for computer access |
| Giesbrecht | 2011 | Experiences with using a pushrim-activated power-assisted wheelchair for community-based occupations: a qualitative exploration | Canada | The purpose of this study was to explore the experience of using a pushrim-activated power-assist wheelchair for community-based occupations of meaning among individuals who use both a manual wheelchair and a power wheelchair. | 8 people aged 33 to 63 (2 female, 6 male) who use both manual and power wheelchairs; 4 of these (all male) with SCI | Focus group | Qualitative description approach | Pushrim-activated power-assist wheelchair use |
| Giroux | 2021 | Enhancing participation while aging with spinal cord injury: applying behaviour change frameworks to develop intervention recommendations | Canada | This study sought to explore the lived experiences of individuals with SCI after their injury, as well as obtain the perspectives of therapists working with SCI patients. | 22 people with SCI (10 female, 12 male); aged at least 45 years and at least 10 years post injury | Semi structured interviews | Deductive extraction based on Knowledge to Action Framework | Ageing with SCI |
| Hammel | 2015 | Environmental barriers and supports to everyday participation: a qualitative insider perspective from people with disabilities | USA | The aim of this study was to describe environmental factors that influence participation of people with disabilities. | 201 people with diverse disabilities across 36 focus groups; number of people with SCI not specified. | Focus group (secondary analysis of qualitative data from 5 projects) | Constant comparison | Environmental barriers and supports |
| Hammell | 2009 | Managing fatigue following spinal cord injury: a qualitative exploration | Canada | The aim of this study was to identify, from the perspectives of people with spinal cord injury (SCI), (a) appropriate components of a fatigue management programme; and (b) important outcomes or indicators of success. | 29 participants including 21 people with SCI (9 female, 12 male); aged 26 to 69 years; 2 family members; 2 assistants; 4 occupational therapists | Focus group | Interpretive data analysis | Fatigue management |
| Hearn | 2018 | Stigma and self-management: an interpretative phenomenological analysis of the impact of chronic recurrent urinary tract infections after spinal cord injury | UK | This study aims to provide useful insights and potential targets for intervention to improve patient-centred care by exploring the subjective understandings and experiences of symptomatic UTIs, if and how these experiences converge and diverge, their management strategies and the psychosocial impact of UTIs on people living with recurrent UTIs after SCI. | 12 people with SCI (6 female, 6 male) aged between 28 and 68 years who had experienced at least 3 UTIs needing antibiotic treatment in the previous 12 months | Semi structured interviews | Interpretative phenomenological analysis | Chronic recurrent urinary tract infections |
| Isaksson | 2007 | To regain participation in occupations through human encounters narratives from women with spinal cord injury | Sweden | The aim of this study was to gain an understanding of how women with spinal cord injury (SCI) experienced human encounters in occupations and how these influenced their participation. | 13 women aged 25 to 61 with cervical or thoracic SCI; 1-10 years post SCI | Semi structured interviews | Paradigmatic analysis of narrative data | Human encounters in occupations |
| Isaksson | 2007 | Social support provides motivation and ability to participate in occupation | Sweden | The aim of the current study was to describe how women with spinal cord injuries perceive the importance of social support for participation in occupation. | 13 women aged 25 to 61 with cervical or thoracic SCI; 1-10 years post SCI | Semi structured interviews | Grounded theory | Social support |
| Jorgensen | 2017 | Negotiating identity and self-image: perceptions of falls in ambulatory individuals with spinal cord injury - a qualitative study | Norway | The aim of this study was to explore and describe experiences and perceptions of falls, risk of falling and fall-related consequences among ambulatory individuals with traumatic SCI. | 15 people (5 female, 10 male) with SCI; aged 23 to 78; 2-34 years post injury; walking at least 75% of the time for mobility needs; fall experience in previous year. | Semi structured interviews | Thematic interpretive content analysis | Falls |
| Kern | 2019 | Understanding the changing health care needs of individuals aging with spinal cord injury | USA | The purpose of this qualitative study was twofold: to explore changing health care needs of individuals aging with SCI and their caregivers and identify environmental supports and barriers to achieving those long-term outcomes. | 41 people with SCI aged greater than 60 years and at least 5 years post injury; 8 non paid family caregivers | Semi structured interviews | Emergent themes and subthemes were identified using naturalistic inquiry | Ageing |
| Lundstrom | 2015 | The purpose of this study was to gain an understanding of participation in everyday occupations through life stories of persons aging with a traumatic sci | Sweden | The purpose of this study was to gain an understanding of participation in everyday occupations through life stories of persons aging with a traumatic SCI. | 8 people with tetraplegia (4 female, 4 male) caused by SCI; aged 48 to 74; at least 10 years post injury | Semi structured interviews | Paradigmatic analysis; inductive approach | Ageing |
| Mattar | 2015 | A qualitative study on the use of personal information technology by persons with spinal cord injury | Canada | The purpose of the present study was to obtain an in-depth perspective of the ways that information technology (IT) is used by community-dwelling persons with SCI. | 10 community dwellers with SCI (7 female, 3 male); aged 20 to 75; self identifying as being regular users of IT | Mixed methods; semi structured interview | Qualitative content analysis | Information technology |
| Newman | 2010 | Evidence-based advocacy: using photovoice to identify barriers and facilitators to community participation after spinal cord injury | USA | The specific aims of this project were to: engage people with SCI in a CBPR (community based participatory research) approach that uses the Photovoice method to gather and analyze data on environmental barriers and facilitators to community participation; create an evidence base of environmental barriers and facilitators to community participation; use the findings of this project as a foundation for evidence-based advocacy. | 10 wheelchair users with SCI (4 female, 6 male); aged 20 to 61; at least 1 year post injury. | Semi structured interviews; photovoice | Identify issues or themes and develop theories that are anchored in the data | Environmental barriers and facilitators |
| Riedman | 2020 | “Earth angels” and parking spots: qualitative perspectives on healthy aging with spinal cord injury | USA | The aim of this study was to understand the subjective experiences of persons aging with SCI in relation to their surrounding social and physical environments, including the impact of barriers and facilitators. | 11 people with SCI (3 female, 8 male); aged at least 45; at least 10 years post injury; community dwelling | Mixed methods; semi structured interviews | Thematic grounded theory approach | Ageing |
| Ripat | 2012 | The role of assistive technology in self-perceived participation | Canada | The purpose of this research was to develop an understanding of how assistive technology factors into self-perceived participation for individuals with spinal cord injuries. | 19 people with SCI (6 female, 13 male); aged from 20 to over 60. | Semi structured interview; focus group; photovoice | Constant comparison | Assistive technology |
| Singh | 2020 | Exploring the causes and impacts of falls among ambulators with spinal cord injury using photovoice: a mixed-methods study | UK | This study explored: (1) fall circumstances experienced by ambulators with spinal cord injury (SCI) over a 6-month period, (2) the impacts of falls-related injuries and fall risk and (3) their preferences/recommendations for fall prevention | 33 ambulators with SCI for full study; 8 (2 female, 6 male) of the 33 participated in interviews; 5 of these (1 female, 4 male) participated in focus group discussion; aged between 45 and 69. | Mixed methods; semi structured interviews; focus group; photovoice | Inductive thematic analysis | Falls |
| Smith | 2015 | The impact of living in a care home on the health and wellbeing of spinal cord injured people | UK | The purpose of this research was to examine the impact of living in a care home environment on the health and wellbeing of people with SCI. | 20 people (5 female, 15 male) with traumatic SCI; aged 21 to 70; who were  living in a care home, or had done so in last 6 months | Semi structured interviews | Inductive thematic analysis | Living in a care home |
| Verdonck | 2011 | Electronic aids to daily living: be able to do what you want | Ireland | The purpose of this study was to explore the experiences and the deeper meaning of living with electronic aids to daily living (EADL) from both user’s and potential user’s perspectives. This study is the first part of a larger study that seeks to explore the experience of using EADL from the user’s perspective. | 15 (4 female, 11, male) people with SCI from levels C3 to C5; aged 20 to 57 years; 1-31 years post discharge from rehabilitation; 8 had EADL and 7 did not | Focus group | Descriptive phenomenological analysis | Electronic aids to daily living |
| Wall | 2020 | Experiences of communication changes following spinal cord injury: a qualitative analysis | Australia | This study seeks to provide a deeper understanding of the challenges faced by individuals with communication changes following cervical SCI, from a biopsychosocial perspective, and elucidate the skills and support required to adjust to these changes in order to live successfully post injury. | 14 community dwelling, non-ventilated people (11 male, 3 female) with cervical level SCI; sustained within past 6 years | Semi structured interviews | Inductive thematic analysis | Voice and communication changes |
| Wangdell | 2013 | Enhanced independence: experiences after regaining grip function in people with tetraplegia | Sweden | The aim of this study was to develop a deeper understanding of how surgical restoration of grip function affects patients' lives, with emphasis not only on activity and participation but also on personal and environmental factors. | 11 people (1 female, 10 male) with tetraplegia due to SCI; aged 22 to 73; who had undergone grip reconstructive surgery | Semi structured interviews | Grounded theory | Surgical restoration of grip focus |
| Ward | 2007 | Occupation-based practice and its relationship to social and occupational participation in adults with spinal cord injury | USA | The purpose of the current study was to explore the social and occupational participation of three individuals with spinal cord injuries and the occupation-based approaches that the participants felt were most helpful in achieving those outcomes. | 3 community dwelling people (2 female, 1 male) with SCI; sustained in the previous 2-5 years; had received occupational therapy services | Semi structured interviews | Thematic analysis Narrative analysis | Occupational therapy services |
| Welk | 2021 | A qualitative assessment of psychosocial aspects that play a role in bladder management after spinal cord injury | USA Canada | Our objective was to gain a better understanding of the relevant psychosocial aspects of bladder management and urinary catheters among a community-dwelling sampling of people living with a SCI. | 34 people with SCI (13 female, 21 male); aged 27 to 69; at least 6 months post injury | Semi structured interviews; focus group | Interpretative phenomenological approach | Bladder management |
| Zinman | 2014 | Evaluation of a community reintegration outpatient program service for community-dwelling persons with spinal cord injury | Canada | The primary objective of this study was to investigate the efficacy of the community reintegration outpatient (CROP)service for community-dwelling individuals with an SCI. | 21 community dwelling people with SCI; less than 3 years post injury;12 people participated in the qualitative component | Mixed methods; semi structured interviews | Inductive content analysis | Community integration outpatient program |
